# Analysis of bone marrow supernatant neutrophil gelatinase‐associated lipocalin and hematological parameters in hematological malignancy

**DOI:** 10.1002/jcla.23253

**Published:** 2020-02-24

**Authors:** Chi‐Hyun Cho, Jaehyung Cha, Eun‐Ah Chang, Myung‐Hyun Nam, Seo‐Jin Park, Hwa Jung Sung, Se Ryeon Lee

**Affiliations:** ^1^ Department of Laboratory Medicine College of Medicine Korea University Ansan Hospital Ansan‐si Korea; ^2^ Medical Science Research Center Korea University Ansan Hospital Ansan‐si Korea; ^3^ Department of Hematology College of Medicine Korea University Ansan Hospital Ansan‐si Korea

**Keywords:** bone marrow, hematological, malignancy, neutrophil counts, neutrophil gelatinase‐associated lipocalin

## Abstract

**Background:**

Neutrophil gelatinase‐associated lipocalin (NGAL) is a urine biomarker related to acute renal injury. Whereas several studies have evaluated NGAL levels in hematological malignancy, using peripheral blood (PB). Recently, bone marrow (BM) NGAL level was reported to be higher than PB NGAL level in individuals with hematological malignancy, suggesting that BM NGAL would reflect BM microenvironment better than PB NGAL. We measured BM NGAL levels in patients with hematological malignancy, comparing those with NGAL levels in normal BM. We evaluated the association of BM NGAL with hematological parameters including neutrophil counts.

**Methods:**

BM samples were collected from 107 patients who underwent BM examination. Immunoassays were used to assess NGAL levels. Data on hematological parameters were collected from medical records. Intergroup comparisons were performed using the Kruskal‐Wallis H test and Pearson chi‐square test. Single and multiple regression analyses were performed to analyze the relationships.

**Results:**

The independent factors that affected the BM NGAL level were neutrophil counts and BM band neutrophil%, while neutrophil count was the main influencing factor. The acute myeloid leukemia (n = 18) and myelodysplastic syndrome (n = 25) groups showed statistically lower BM NGAL levels than patients with normal BM. The myeloproliferative neoplasm group (n = 34) showed higher BM NGAL levels than patients with normal BM, but this difference was not statistically significant. Neutrophil counts and BM band neutrophil% showed intergroup patterns similar to those of BM NGAL levels.

**Conclusion:**

BM NGAL was related to neutrophil count and BM band neutrophil%, showing different levels according to hematological malignant disease entities.

## 1 INTRODUCTION

Recently, chronic inflammation has been hypothesized to lead to the development of hematological malignancy.^1^, ^2^, ^3^ According to this hypothesis, the analysis of inflammation makers will help elucidate the mechanism of hematological malignancy.^1^

Neutrophil gelatinase‐associated lipocalin (NGAL) is known as a biomarker related to acute renal injury, but was also referred to as an adipokine due to its association with inflammation.^4^, ^5^ The association of NGAL with inflammation has been demonstrated in several studies.^5^ In addition, the NGAL level has been reported to increase in individuals with hematological malignancies such as chronic myeloid leukemia (CML), polycythemia vera (PV), and essential thrombocythemia (ET).^5^, ^6^, ^7^, ^8^

In previous studies evaluating the NGAL levels in patients with hematological malignancies, the samples examined were mostly peripheral blood (PB).^5^, ^6^, ^7^ Although a previous study was conducted using human bone marrow (BM) samples, the only disease entity examined in that study was acute myeloid leukemia (AML).^9^

Another study reported that the level of BM NGAL was significantly higher than that of PB NGAL in individuals with hematological malignancy, suggesting that BM NGAL would reflect the BM microenvironment better than PB NGAL.^10^ Hematological malignancy develops in the BM, while neutrophilic precursors as the major source of NGAL synthesis exist mostly in the BM.^5^ Accordingly, the accurate analysis of NGAL according to the hematological malignancy requires the measurement of BM NGAL rather than PB NGAL.^10^

Meanwhile, the PB NGAL level was reported to show a statistically significant association with the neutrophil count. Hence, BM NGAL might also show a significant relation with the neutrophil count; however, further study is warranted to confirm this speculation.^10^

This study aimed to measure the NGAL levels in BM samples of patients who underwent BM examination for the diagnosis of hematological malignancies and evaluate whether BM NGAL has a significant association with hematological parameters including neutrophil count. In addition, we aimed to compare the NGAL levels in patients with AML, myelodysplastic syndrome (MDS), myeloid proliferative neoplasm (MPN), and plasma cell neoplasm (PCN) with those in control.

## 2 MATERIALS AND METHODS

### 2.1 Sample collection and preparation

The study was approved by the Institutional Review Board of the Korea University Ansan Hospital and was performed according to the Declaration of Helsinki. Patients were enrolled from June 2015 to July 2019. Informed consent was obtained from all patients who participated in the study (n = 107). Aliquots of leftover BM aspirate samples were collected from 107 patients who underwent BM examination for the diagnosis of hematological malignancies at the Korea University Ansan Hospital. Patients were classified into disease groups based on the World Health Organization diagnostic criteria for MPN, AML, MDS, and PCN in the BM smear and pathology review. The control group (n = 14) comprised patients with lymphoma without BM involvement (n = 12) or those with normocellular marrow without hematological malignancy in the BM smear and pathology review (n = 2).^9^ None of the patients, including those in the control group, had symptoms of active infections, inflammatory diseases, or kidney failure.^6^ BM aspirate supernatants were collected from tubes containing ethylenediaminetetraacetic acid after centrifugation (2399 *g*, 10 minutes) and stored at −80°C until analysis. The NGAL levels in BM aspirate samples were analyzed using an immunoassay.

### 2.2 Clinical data collection

Baseline demographic data and hematological parameters such as hemoglobin, white blood cell (WBC) count, neutrophil and platelet count, C‐reactive protein (CRP), Chronic Kidney Disease Epidemiology Collaboration equation (CKD‐EPI), BM cellularity, myeloid:erythroid (M:E) ratio, and BM cell% (BM cell count on BM aspiration slides) were collected from electronic medical records. BM cell% included BM blast%, BM promyelocyte%, BM myelocyte%, BM metamyelocyte%, BM band neutrophil%, and BM neutrophil%.

### 2.3 Neutrophil gelatinase‐associated lipocalin immunoassay

Neutrophil gelatinase‐associated lipocalin levels were measured using a commercially available NGAL particle‐enhanced turbidimetric immunoassay kit (BioPorto Diagnostics) used on a Cobas 8000 automation platform (Roche Diagnostics), as assessment of BM supernatant using the immunoassay kit (BioPorto Diagnostics) was validated in a previous study.^10^ BM supernatant samples were not diluted. The automated sequence involved a 5‐min incubation of 3 μL of sample and 150 μL of reaction buffer, followed by another 5‐min incubation of 50 μL of immunoparticle suspension. NGAL concentration was calculated from changes in absorbance, based on a calibration curve, prepared using the calibration results of known concentrations (0‐3000 ng/mL). Measurements were performed in duplicate and the results were averaged.

### 2.4 Statistical analyses

Quantitative data were presented as median (quartile [Q]1,Q 3). To ascertain whether or not the data were normally distributed, the Shapiro‐Wilk test was applied. Since the data did not meet the criteria of a normal distribution, the non‐parametric Kruskal‐Wallis H test was applied for the intergroup comparisons. If a significant difference (a *P*‐value <.05) was found in the initial analysis, the pairwise test was performed using adjusted *P*‐value after Bonferroni correction. The Pearson chi‐square test was used for intergroup comparisons of categorical data. A simple regression analysis was performed to analyze the relationship of BM NGAL levels with age, gender, and each of the hematological parameters. A multiple regression analysis was then performed to analyze the relationship of BM NGAL levels with other parameters simultaneously, while stepwise regression was used as a model selection procedure. The predictive accuracy of the multiple regression model was assessed using adjusted *R* ^2^ values. Statistical significance was set at *P* < .05. The statistical analyses were performed using SPSS (version 20.0; IBM SPSS Statistics).

## 3 RESULTS

### 3.1 Patient characteristics

Bone marrow supernatant samples were collected from 107 patients with a median age of 63 (range: 22‐89) years; approximately 60.7% (65/107) of the patients were men and 39.3% (42/107) were women. The underlying diagnoses were MPN (n = 34), AML (n = 18), MDS (n = 25), and PCN (n = 16); the control group comprised 14 patients. Cases (n = 34) of MPN comprised CML (n = 12), PV (n = 12), ET (n = 6), primary myelofibrosis (PMF) (n = 3), and MPN‐unclassifiable (MPN‐U) (n = 1). All of the 107 patients were at the initial diagnosis stage. Table 1 presents the age, gender, and hematological parameters of the patients according to disease entities of hematological malignancy.

**Table 1:** Patients’ demographic features and laboratory parameters in each group of hematological malignancy, including NGAL (n = 107)

	MPN^a^	AML	MDS	PCN	Control^b^	*P*‐value*
Patients	34	18	25	16	14	
Age	57 (48, 64)	59 (43, 75)	75 (60, 82)	68 (57, 76)	61 (44, 71)	.004
Gender	16 males 18 females	15 males 3 females	11 males 14 females	11 males 5 females	12 males 2 females	.009
Hb (g/L)	127 (105, 170)	75 (57, 86)	86 (70, 91)	114 (91, 138)	134 (119, 151)	<.0001
WBC count (10^9^/L)	14.57 (10.37, 80.17)	3.37 (1.53, 8.20)	2.38 (1.58, 3.96)	5.63 (4.53, 6.87)	6.66 (4.59, 7.86)	<.0001
Neutrophil count (10^9^/L)	11.06 (7.21, 53.86)	0.74 (0.21, 1.80)	1.05 (0.55, 1.70)	3.30 (2.34, 4.36)	4.10 (2.52, 4.90)	<.0001
Platelet count (10^9^/L)	616 (382, 887)	37 (24, 110)	78 (32, 216)	195 (132, 231)	301 (237, 319)	<.0001
M:E ratio	2.5 (1.5, 8.5)	13.2 (3.2, 58.3)	1.4 (0.7, 3.2)	3.2 (2.1, 6.5)	1.7 (1.3, 2.1)	<.0001
BM blast %	1.0 (0.6, 2.1)	69.2 (43.2, 87.1)	4.4 (1.9, 7.3)	0.9 (0.3, 1.4)	0.8 (0.5, 1.3)	<.0001
BM promyelocyte %	3.2 (1.8, 7.7)	1.0 (0.4, 7.2)	2.5 (1.2, 6.8)	0.6 (0.1, 2.5)	3.3 (0.8, 5.0)	.018
BM myelocyte %	13.7 (9.2, 17.0)	2.5 (1.3, 3.5)	8.3 (6.2, 15.5)	5.0 (3.0, 10.8)	9.7 (7.8, 11.7)	<.0001
BM metamyelocyte %	4.8 (3.4, 8.4)	1.2 (0.2, 2.0)	2.8 (1.8, 5.6)	2.6 (0.6, 5.9)	3.4 (1.8, 4.8)	<.0001
BM band neutrophil %	8.8 (7.2, 13.2)	0.8 (0, 1.7)	4.8 (1.7, 7.9)	4.7 (2.2, 9.5)	7.9 (5.6, 10.7)	<.0001
BM neutrophil%	20.5 (16.8, 25.9)	0.7 (0.6, 4.8)	8.6 (6.7, 18.2)	13.9 (7.4, 30.0)	20.7 (15.3, 21.8)	<.0001
BM cellularity	85 (60, 95)	90 (44, 95)	35 (20, 75)	50 (26, 75)	37 (25, 45)	<.0001
CRP (mg/dL)	0.108 (0.050, 0.650)	6.990 (0.686, 11.525)	0.838 (0.280, 2.701)	0.214 (0.108, 0.919)	0.140 (0.067, 0.182)	<.0001
CKD‐EPI (mL/min/1.73m^2^)	91.8 (79.7 ~ 108.6)	89.8 (69.4 ~ 99.9)	80.0 (57.5 ~ 95.3)	82.5 (50.1 ~ 89.3)	92.6 (85.6 ~ 108.8)	.023
NGAL (ng/mL)	511.03 (288.40, 1389.99)	15.60 (9.00, 43.39)	59.30 (25.63, 100.63)	113.45 (72.46, 170.61)	185.78 (159.94, 257.26)	<.0001

Note

Quantitative data are presented as median (quartile [Q] 1,Q 3).

Abbreviations: AML, acute myeloid leukemia; BM, bone marrow; CKD‐EPI, Chronic Kidney Disease Epidemiology Collaboration equation; CML, chronic myeloid leukemia; CRP, C‐reactive protein; ET, essential thrombocythemia; Hb, hemoglobin; MDS, myelodysplastic syndrome; M:E, myeloid: erythroid; MPN, myeloproliferative neoplasm; MPN‐U, MPN‐unclassifiable; NGAL, neutrophil gelatinase‐associated lipocalin; PCN, plasma cell neoplasm; PMF, primary myelofibrosis; PV, polycythemia vera; WBC, white blood cell.

a MPN included CML (n = 12), PV (n = 12), ET (n = 6), PMF (n = 3), and MPN‐U (n = 1).

b the control group comprised patients with lymphoma without bone marrow involvement (n = 12) or described as normocellular marrow without hematological malignancy in the bone marrow smear and pathology review (n = 2).

* For categorical data (gender variable), the Pearson chi‐square test was performed and for numerical data, Kruskal‐Wallis H test was performed.

### 3.2 Comparison of numerical data among groups

**Figure 1:**
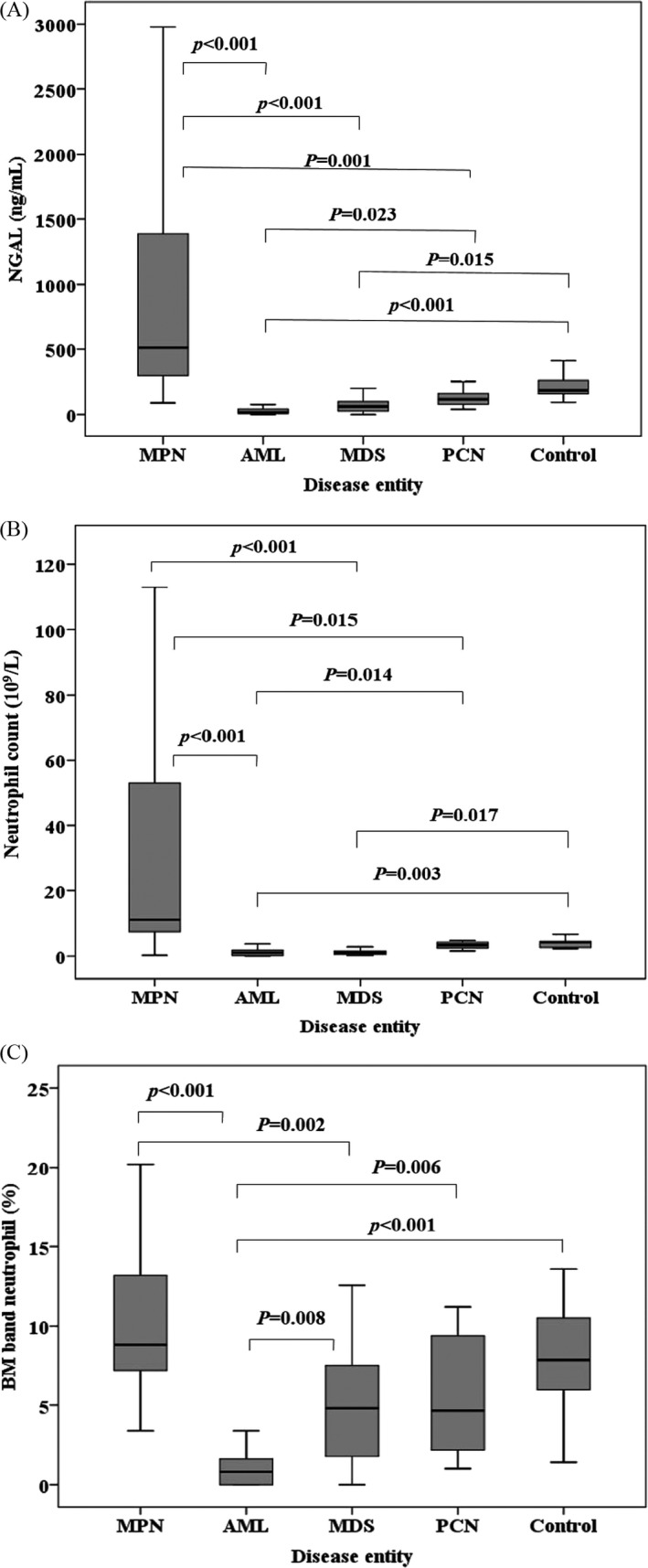
Comparison of NGAL levels (A), neutrophil counts (B), and BM band neutrophil% (C) in patients with hematological malignancy and the control group. The control group comprised patients with normal bone marrow. (A) NGAL levels in the AML and MDS groups are statistically lower than those in the control group. NGAL levels in the AML group are statistically lower than those in the MPN and PCN groups. NGAL levels in the MDS group are statistically lower than those in the MPN group. NGAL levels in the PCN group are statistically lower than those in the MPN group. (B) Neutrophil counts in the AML and MDS groups are statistically lower than those in the control group. Neutrophil counts in the AML group are statistically lower than those in the MPN and PCN groups. Neutrophil counts in the MDS group are statistically lower than those in the MPN group. Neutrophil counts in the PCN group are statistically lower than those in the MPN group. (C) BM band neutrophil% in the AML group is statistically lower than that in the MPN, MDS, PCN, and control groups. BM band neutrophil% in the MDS group is statistically lower than that in the MPN group. Abbreviations: AML, acute myeloid leukemia; BM, bone marrow; MDS, myelodysplastic syndrome; MPN, myeloproliferative neoplasm; NGAL, neutrophil gelatinase‐associated lipocalin; PCN, plasma cell neoplasm

For categorical data (gender variable), Pearson's chi‐square test was performed, and for numerical data, the Kruskal‐Wallis H test was performed to assess significant differences between groups (Table 1). Seventeen variables showed statistically significant differences among the five groups. For numerical data, the statistically significant results of pairwise comparison test between groups are shown in Figure 1 and Table S1.

### 3.3 Analysis of the relationship of BM NGAL levels with age, gender, and hematological parameters

Simple regression analysis identified 13 significant predictors of BM NGAL levels (Table 2). Next, a multiple regression analysis of the 13 predictors with stepwise regression was performed, leaving only two independent variables **(**neutrophil count and BM band neutrophil%). The multiple regression model was as follows (Table 3):

**Table 2:** Simple regression analysis of bone marrow neutrophil gelatinase‐associated lipocalin levels with age, hematological parameters, and CRP in hematological cancers

Clinical parameters	*R* ^2^	*P‐*value
Age	.080	.003*
Gender	.011	.289
Hb (g/L)	.055	.015*
WBC (10^9^/L)	.389	<.0001*
Neutrophil count (10^9^/L)	.543	<.0001*
Platelet count (10^9^/L)	.075	.004*
CRP (mg/dL)	.026	.115
M:E ratio	.002	.675
BM blast %	.055	.015*
BM promyelocyte %	.119	<.0001*
BM myelocyte %	.288	<.0001*
BM metamyelocyte %	.250	<.0001*
BM band neutrophil %	.266	<.0001*
BM neutrophil%	.116	<.0001*
BM cellularity	.146	<.0001*
CKD‐EPI (mL/min/1.73m^2^)	.042	.036*

Abbreviations: BM, bone marrow; CKD‐EPI, Chronic Kidney Disease Epidemiology Collaboration equation; CRP, C‐reactive protein; Hb, hemoglobin; M:E, myeloid: erythroid; WBC, white blood cell.

* Statistically significant (*P* < .05).

**Table 3:** Regression analysis of the relationship of BM NGAL levels with hematological parameters in hematological malignancies

		Coefficient (95% CI)	*t*‐value	*P*‐value	Adj *R* ^2^
Model	Constant	−33.596 (−154.501 ~ 87.310)	‐0.551	.583	.615
	Neutrophil count	18.230 (14.578 ~ 21.882)	9.905	<.0001	
	BM band neutrophil%	26.486 (8.471 ~ 44.501)	2.917	.004	

Abbreviations: Adj, adjusted; BM, bone marrow; CI, confidence interval; NGAL, neutrophil gelatinase‐associated lipocalin.

* Statistically significant (*P* < .05).

(Model) BM NGAL = 18.230 × neutrophil count + 26.486 × BM band neutrophil%−33.596

In this model, the significant independent variables were neutrophil count (*P* < .0001) and BM band neutrophil% (*P* = .004).

### 3.4 Neutrophil gelatinase‐associated lipocalin levels in hematological malignant diseases compared to control

The median (Q1, Q3) of NGAL levels according to the disease entities of hematological malignancy are presented in Table 1. The median (Q1, Q3) of NGAL levels in control group was 185.78 (159.94, 257.26) ng/mL. MPN (n = 34) showed the highest NGAL levels [median (Q1, Q3); 511.03 (288.40, 1389.99) ng/mL]. In the MPN group, the CML group (n = 12) showed higher NGAL levels [1740.15 (890.61, 2312.09) ng/mL] than the PV groups (n = 12) [451.55 (276.54, 627.69) ng/mL]. The MPN group showed statistically higher NGAL levels than the AML or MDS groups (Figure 1A).

Acute myeloid leukemia showed the lowest NGAL levels [15.60 (9.00, 43.39) ng/mL]. AML and MDS groups showed statistically significant lower NGAL levels than the control group (Figure 1A). MPN group showed higher NGAL levels than the control group, but the difference was not statistically significant (*P* = .289). PCN group showed lower NGAL levels than the control group, but the difference was not statistically significant (*P* = 1.000).

### 3.5 Neutrophil counts in hematological malignant diseases compared with those in control

The median (Q1, Q3) of the neutrophil counts in the control group was 4.10 (2.52, 4.90) ×10^9^/L. MPN (n = 34) showed the highest neutrophil counts [median (Q1, Q3); 11.06 (7.21, 53.86) ×10^9^/L]. The MPN group showed statistically higher neutrophil counts than the AML, MDS, and PCN groups (Table 1; Figure 1B).

Acute myeloid leukemia showed the lowest neutrophil counts [0.74 (0.21, 1.80) ×10^9^/L]. The AML and MDS groups showed statistically significant lower neutrophil counts than the control group. The MPN group showed higher neutrophil counts than the control group, but the difference was not statistically significant (*P* = .148). The PCN group showed lower neutrophil counts than the control group, but the difference was not statistically significant (*P* = 1.000).

### 3.6 Bone marrow band neutrophil% in hematological malignant diseases compared to control

The median (Q1, Q3) of BM band neutrophil% in the control group was 7.9 (5.6, 10.7) %. MPN (n = 34) showed the highest BM band neutrophil% [median (Q1, Q3); 8.8 (7.2, 13.2) %]. The MPN group showed a statistically higher BM band neutrophil% than the AML or MDS groups (Table 1; Figure 1C).

Acute myeloid leukemia showed the lowest BM band neutrophil% [0.8 (0, 1.7) %]. The AML group showed a statistically significant lower BM band neutrophil% than the control group. The MPN group showed a higher BM band neutrophil% than the control group, but the difference was not statistically significant (*P* = 1.000). The PCN group showed a lower BM band neutrophil% than the control group, but the difference was not statistically significant (*P* = 1.000).

## 4 DISCUSSION

In this study, we measured the BM NGAL levels and evaluated if BM NGAL has a significant association with hematological parameters including neutrophil count. In addition, we compared the BM NGAL levels in the MPN, AML, MDS, and PCN groups with those in the control group (normal BM).

The number of parameters showing statistically significant differences among groups was 17 (Table 1). However, stepwise regression analysis showed that the independent factors affecting BM NGAL were neutrophil count (*P* < .0001) and BM band neutrophil% (*P* = .004), while the multiple regression model including the two factors showed an adjusted *R* ^2^ of 61.5% (Table 3). This finding suggests that these two factors could account for 61.5% of the BM NGAL level. A simple regression analysis evaluating the relationship between BM NGAL and each factor showed that neutrophil count had the highest *R* ^2^ (.543, *P* < .0001; Table 2). The reason why neutrophil count is the most influencing factor for BM NGAL levels is probably because mature neutrophils secrete NGAL. While a previous study showed that the PB NGAL level has a statistically significant association with the neutrophil count, our study showed that the BM NGAL level also had a significant association with the neutrophil count.^10^ However, our study showed that BM band neutrophil% is also a statistically significant influencing factor for BM NGAL (Table 3), although the *R* ^2^ (.266, *P* < .0001; Table 2) of BM band neutrophil% was lower than that of neutrophil count (0.543, *P* < .0001; Table 2). Given that NGAL is synthesized during the early stage of neutrophil maturation and secreted by mature neutrophils, our result suggests that NGAL secretion might begin during the stage of band neutrophils and increase during mature neutrophils, as NGAL secretion affects the NGAL levels.^5^ However, a future study is warranted to confirm whether NGAL secretion begins during the stage of band neutrophils, which are the earliest neutrophilic precursors.

Myeloid proliferative neoplasm groups had higher platelet counts than other groups (Table 1).In particular, MPN groups had statisctially higher platelet counts than AML, MDS, and PCN groups (Table S1). Likewise, MPN groups showed statistically higher BM NGAL levels than AML, MDS, and PCN groups (Figure 1A). Clearly, platelet count was one of independent variables influencing BM NGAL (*P* = .004; Table 2). However, the explanatory power of the platelet count was very small (*R* ^2^ = .075; Table 2). Above all things, multiple regression analysis with stepwise regression as a model selection procedure showed that platelet count was removed and only neutrophil count and BM band neutrophil% remained as statistically significant variables influencing NGAL (Tables 2 and 3).

The AML group showed statistically lower NGAL levels than the control group, and this finding is similar to the results of a previous study, which measured the NGAL level in BM samples of AML patients.^9^ In addition, NGAL levels in the AML group were the lowest among those in other disease entities (Figure 1A). The reason why NGAL levels in AML group was low would be probably because leukemic blasts suppressed trilineage hematopoietic precursors including neutrophilic precursors and mature neutrophils.

The NGAL levels in MDS groups were statistically lower than that in the control group, which, to the best of our knowledge, has not been reported previously (Figure 1A). Considering the pathogenesis of MDS, this finding may be attributable to the dysregulation of NGAL synthesis or secretion in immature neutrophilic precursors or mature neutrophils, regardless of the increase in the number of immature neutrophilic precursors and mature neutrophils in the BM.^11^

The MPN group showed higher NGAL levels than the control group, and this difference was not statistically significant (*P* = .289; Figure 1A). The CML and PV groups, the MPN subgroups, also showed higher NGAL levels than the control group; this difference was also not statistically significant (*P* = .54 and *P* = 1.000, respectively). However, in previous studies, the CML and PV groups showed statistically higher NGAL levels than the control group.^6^, ^7^ The MPN group having higher NGAL levels than the control group (normal BM) may be attributable to the increase in the levels of neutrophilic precursors and mature neutrophils in BM, which leads to an increase in the synthesis and secretion of NGAL.

The PCN group showed lower NGAL levels than the control group; this difference was not statistically significant (*P* = 1.000). The PCN group had lower NGAL levels than the control group probably because the neutrophilic precursors and mature neutrophils were suppressed by plasma cells.

Expectedly, neutrophil counts and BM band neutrophil%, the two independent factors affecting BM NGAL showed intergroup patterns similar to those of BM NGAL levels (Figure 1). Also for both of the factors, the AML group showed statistically lower levels than the control group, while the MDS group showed lower levels and the MPN group showed higher levels than the control group.

In our study, the control group comprised patients with lymphoma without bone marrow involvement or those having normocellular marrow without hematological malignancy in the BM smear and pathology review, because BM examination is an invasive procedure and not commonly performed in healthy persons. Nevertheless, the control group in our study had normal hemoglobin level, WBC counts, neutrophil counts, platelet counts, and CRP level and had no symptoms of active infections, inflammatory diseases, or kidney failure (Table 1).

In conclusion, NGAL was measured using BM supernatant of patients confirmed as having hematological malignancy in the BM smear and pathology review. The independent factors that affected BM NGAL level with statistical significance were neutrophil count and BM band neutrophil%, while neutrophil count was the main influencing factor. When comparing BM NGAL levels according to the disease group, the AML and MDS groups showed statistically lower BM NGAL levels than the control group (normal BM). The MPN group showed higher BM NGAL levels than the control group, although this difference was not statistically significant. Neutrophil counts and BM band neutrophil% showed intergroup patterns similar to that of BM NGAL levels. BM NGAL was related to neutrophil count and BM band neutrophil%, showing different levels according to hematological malignant disease entities.

## References

[1] Takizawa H , Manz MG . Impact of inflammation on early hematopoiesis and the microenvironment. Int J Hematol. 2017;106(1):27‐33. 28560577 10.1007/s12185-017-2266-5

[2] Kristinsson SY , Bjorkholm M , Hultcrantz M , Derolf AR , Landgren O , Goldin LR . Chronic immune stimulation might act as a trigger for the development of acute myeloid leukemia or myelodysplastic syndromes. J Clin Oncol. 2011;29(21):2897‐2903. 21690473 10.1200/JCO.2011.34.8540 PMC3138717

[3] Kristinsson SY , Landgren O , Samuelsson J , Bjorkholm M , Goldin LR . Autoimmunity and the risk of myeloproliferative neoplasms. Haematologica. 2010;95(7):1216‐1220. 20053870 10.3324/haematol.2009.020412 PMC2895049

[4] Abella V , Scotece M , Conde J , et al. The potential of lipocalin‐2/NGAL as biomarker for inflammatory and metabolic diseases. Biomarkers. 2015;20(8):565‐571. 26671823 10.3109/1354750X.2015.1123354 PMC4819811

[5] Chakraborty S , Kaur S , Guha S , Batra SK . The multifaceted roles of neutrophil gelatinase associated lipocalin (NGAL) in inflammation and cancer. Biochim Biophys Acta. 2012;1826(1):129‐169. 22513004 10.1016/j.bbcan.2012.03.008 PMC3362670

[6] Allegra A , Alonci A , Bellomo G , et al. Increased serum levels of neutrophil gelatinase‐associated lipocalin in patients with essential thrombocythemia and polycythemia vera. Leuk Lymphoma. 2011;52(1):101‐107. 21133718 10.3109/10428194.2010.531413

[7] Villalva C , Sorel N , Bonnet ML , et al. Neutrophil gelatinase‐associated lipocalin expression in chronic myeloid leukemia. Leuk Lymphoma. 2008;49(5):984‐988. 18464118 10.1080/10428190801942360

[8] Leng X , Lin H , Ding T , et al. Lipocalin 2 is required for BCR‐ABL‐induced tumorigenesis. Oncogene. 2008;27(47):6110‐6119. 18663364 10.1038/onc.2008.209 PMC2756829

[9] Yang WC , Lin PM , Yang MY , et al. Higher lipocalin 2 expression may represent an independent favorable prognostic factor in cytogenetically normal acute myeloid leukemia. Leuk Lymphoma. 2013;54(8):1614‐1625. 23150981 10.3109/10428194.2012.749402

[10] Cho CH , Yoon J , Kim DS , Kim SJ , Sung HJ , Lee SR . Association of peripheral blood neutrophil gelatinase‐associated lipocalin levels with bone marrow neutrophil gelatinase‐associated lipocalin levels and neutrophil count in hematologic malignancy. J Clin Lab Anal. 2019;33(6):e22920. 31090234 10.1002/jcla.22920 PMC6642308

[11] Rankin EB , Narla A , Park JK , Lin S , Sakamoto KM . Biology of the bone marrow microenvironment and myelodysplastic syndromes. Mol Genet Metab. 2015;116(1–2):24‐28. 26210353 10.1016/j.ymgme.2015.07.004 PMC4618471

