# Supplementary material for: Analysis of bone marrow supernatant neutrophil gelatinase‐associated lipocalin and hematological parameters in hematological malignancy
Source: J Clin Lab Anal. 2020 Feb 24;34(6):e23253. doi: 10.1002/jcla.23253 (PMC7307352; doi:10.1002/jcla.23253)
Supplement: Supplementary file 1 [file JCLA-34-e23253-s001.docx]

**Supplementary table 1**. Pairwise comparison analysis between groups for numerical variables (besides neutrophil counts, BM band neutrophil% and NGAL).

| Variable | Comparison groups | *P*-value^*^ |
| --- | --- | --- |
| age | MPN vs. MDS | 0.004 |
| Hb | MPN vs. AML | <0.0001 |
|  | MPN vs. MDS | <0.0001 |
|  | AML vs. PCN | 0.002 |
|  | AML vs. Control | <0.0001 |
|  | MDS vs. PCN | 0.027 |
|  | MDS vs. Control | <0.0001 |
| WBC count | MPN vs. AML | <0.0001 |
|  | MPN vs. MDS | <0.0001 |
|  | MPN vs. PCN | 0.007 |
|  | MPN vs. Control | 0.047 |
| Platelet counts | MPN vs. AML | <0.0001 |
|  | MPN vs. MDS | <0.0001 |
|  | MPN vs. PCN | 0.002 |
|  | AML vs. PCN | 0.042 |
|  | AML vs. Control | <0.0001 |
|  | MDS vs. Control | 0.035 |
| M:E ratio | MPN vs. AML | 0.039 |
|  | AML vs. MDS | <0.0001 |
|  | AML vs. Control | <0.0001 |
| BM blast% | MPN vs. AML | <0.0001 |
|  | MPN vs. MDS | 0.009 |
|  | AML vs. MDS | 0.010 |
|  | AML vs. PCN | <0.0001 |
|  | AML vs. Control | <0.0001 |
|  | MDS vs. PCN | 0.003 |
|  | MDS vs. Control | 0.013 |
| BM promyelocyte% | MPN vs. PCN | 0.011 |
| BM myelocyte% | MPN vs. AML | <0.0001 |
|  | MPN vs. PCN | 0.001 |
|  | AML vs. MDS | <0.0001 |
|  | AML vs. Control | 0.004 |
| BM metamyelocyte% | MPN vs. AML | <0.0001 |
|  | AML vs. MDS | 0.010 |
|  | AML vs. Control | 0.028 |
| BM band neutrophil% | MPN vs. AML | <0.0001 |
|  | MPN vs. MDS | 0.002 |
|  | AML vs. MDS | 0.008 |
|  | AML vs. PCN | 0.006 |
|  | AML vs. Control | <0.0001 |
| BM cellularity | MPN vs. MDS | <0.0001 |
|  | MPN vs. PCN | 0.020 |
|  | MPN vs. Control | <0.0001 |
|  | AML vs. MDS | 0.009 |
|  | AML vs. Control | 0.006 |
| CRP | MPN vs. AML | <0.0001 |
|  | AML vs. Control | <0.0001 |
|  | MDS vs. Control | 0.043 |

^*^, adjusted *P*-value after Bonferroni correction.

**Abbreviations**: AML, acute myeloid leukemia; BM, bone marrow; CML, chronic myeloid leukemia; CRP, C-reactive protein; Hb, hemoglobin; MDS, myelodysplastic syndrome; M:E, myeloid: erythroid; MPN, myeloproliferative neoplasm; NGAL, neutrophil gelatinase-associated lipocalin; PCN, plasma cell neoplasm; WBC, white blood cell.
